# NKG2D and Its Ligand MULT1 Contribute to Disease Progression in a Mouse Model of Multiple Sclerosis

**DOI:** 10.3389/fimmu.2019.00154

**Published:** 2019-02-06

**Authors:** Laurine Legroux, Ana Carmena Moratalla, Cyril Laurent, Gabrielle Deblois, Sandrine L. Verstraeten, Nathalie Arbour

**Affiliations:** Department of Neurosciences Université de Montréal, Montreal, QC, Canada

**Keywords:** T lymphocytes, central nervous system, autoimmune disease, neuroinflammation, NK cell lectin-like receptor subfamily

## Abstract

NKG2D is an activating receptor expressed on the surface of immune cells including subsets of T lymphocytes. NKG2D binds multiple ligands (NKG2DL) whose expression are differentially triggered in a cell type and stress specific manner. The NKG2D-NKG2DL interaction has been involved in autoimmune disorders but its role in animal models of multiple sclerosis (MS) remains incompletely resolved. Here we show that NKG2D and its ligand MULT1 contribute to the pathobiology of experimental autoimmune encephalomyelitis (EAE). MULT1 protein levels are increased in the central nervous system (CNS) at EAE disease peak; soluble MULT1 is elevated in the cerebrospinal fluid of both active and passive EAE. We establish that such soluble MULT1 enhances effector functions (e.g., IFNγ production) of activated CD8 T lymphocytes from wild type but not from NKG2D-deficient (*Klrk1*^−/−^*)* mice *in vitro*. The adoptive transfer of activated T lymphocytes from wild type donors induced a significantly reduced EAE disease in *Klrk1*^−/−^ compared to wild type (*Klrk1*^+/+^) recipients. Characterization of T lymphocytes infiltrating the CNS of recipient mice shows that donor (CD45.1) rather than endogenous (CD45.2) CD4 T cells are the main producers of key cytokines (IFNγ, GM-CSF). In contrast, infiltrating CD8 T lymphocytes include mainly endogenous (CD45.2) cells exhibiting effector properties (NKG2D, granzyme B and IFNγ). Our data support the notion that endogenous CD8 T cells contribute to passive EAE pathobiology in a NKG2D-dependent manner. Collectively, our results point to the deleterious role of NKG2D and its MULT1 in the pathobiology of a MS mouse model.

## Introduction

Multiple sclerosis (MS) is an inflammatory disease of the central nervous system (CNS) characterized by demyelination, axonal loss, activation of glial cells, and infiltration of immune cells, such as macrophages and lymphocytes (1, 2). Although it is well established that the immune system participates in tissue destruction characteristic of MS, the contribution of specific immune mediators to the MS pathobiology has not been fully elucidated (2).

NKG2D is an activating or co-activating receptor expressed on several immune effector cells. Most human CD8 T lymphocytes and activated mouse CD8 T lymphocytes exhibit NKG2D on their surface as well as a small subpopulation of activated CD4 T lymphocytes in both species (3). NKG2D can recognize multiple ligands (NKG2DL); they include Rae-1 (α, β, γ, δ, and ε), H60 (a, b, c) and MULT1 in mice; whereas ULBP (1, 2, 3, 4, 5, and 6) and MIC (A and B) are the human NKG2DL (4). The expression of NKG2DL are regulated at multiple levels (transcription, post-transcription, post-translation, etc.) and vary according to cell type, activation and environmental cues (5–7). Although weakly expressed under normal physiological conditions, NKG2DL are strongly induced in response to stress such as inflammation or infection (4, 6).

Shedding of NKG2DL by tumor cells has been reported in both humans and mice (8). Specific soluble murine and human NKG2DL can trigger NKG2D internalization on effector immune cells and consequently contribute to tumor immune evasion (8). In contrast, soluble MULT1, the murine NKG2DL exhibiting the highest affinity for NKG2D (9), can augment NK cell activation and increase tumor rejection (10). Moreover, soluble MICA, a human NKG2DL, can enhance NK cell functions *in vitro* (11). Soluble NKG2DL have been detected in the serum of patients affected by autoimmune diseases including MS (12–15); it is not fully understood, however, if and how these molecules impact autoimmune pathological processes.

Several studies have suggested that NKG2D and its ligands play a role in the pathobiology of MS. We have previously shown that multiple NKG2DL are detectable at the protein level on human oligodendrocytes in primary cultures (16). We demonstrated that disruption of the NKG2D-NKG2DL interaction inhibits killing of human oligodendrocytes mediated by activated human immune effectors including CD8 T lymphocytes (16). We detected oligodendrocytes expressing MICA/B in post-mortem MS tissues and CD8 T lymphocytes in close proximity to these MICA/B-expressing cells (16). Notably, Ruck and colleagues showed that CD4 T lymphocytes carrying NKG2D are enriched in the blood, cerebrospinal fluid and post-mortem brain lesions of MS patients compared to control donors especially during relapses (17). Whether NKG2D plays a role in MS pathobiology remains to be established.

Experimental autoimmune encephalomyelitis (EAE) is the most commonly used rodent model to investigate this neuroinflammatory disease as it recapitulates multiple immunopathological features of MS (18). Studies by different groups support the notion that NKG2D participates in EAE immunopathobiology. The group of Raulet assessed the susceptibility of NKG2D-deficient (*Klrk1*^−/−^) mice to active EAE induction. They observed that using a suboptimal dose of the encephalitogenic myelin peptide MOG_35−55_, disease was slightly reduced in *Klrk1*^−/−^ compared to wild type mice; such difference was not observed when optimal dose of MOG was injected (19). Wiendl's group showed that blocking NKG2D before, but not after, disease onset diminished active EAE disease severity and migration into the CNS of both CD4 and CD8 T lymphocytes (17). However, it remains unknown whether NKG2D acts via additional mechanisms in EAE pathobiology.

Here, we investigated the role of NKG2D and its ligands in the active and passive EAE models. We establish that one specific NKG2DL, MULT1, is significantly upregulated at the protein level in the spinal cord at the disease peak. Notably during EAE, the cleaved form of MULT1 is elevated not only in this organ but also in the cerebrospinal fluid. We show that soluble MULT1 increases the effector properties of CD8 T lymphocytes *in vitro*. Finally, we demonstrate using the adoptive model of EAE that endogenous T lymphocytes expressing NKG2D contribute to disease progression. Indeed, the adoptive transfer of wild type activated T lymphocytes triggers a significantly less severe disease and fewer CNS-infiltrated T lymphocytes in *Klrk1*^−/−^ compared to *Klrk1*^+/+^ recipients. Overall, our results suggest that NKG2D and its ligands play a role in the pathobiology of MS mouse model.

## Materials and Methods

### Animals

Wild type (WT) C57BL/6J (B6-CD45.2), B6.SJL-*Ptprc*^*a*^ *Pepc*^*b*^/BoyJ (B6-CD45.1), and *Klrk1*^−/−^ (B6.Cg-*Klrk1*^*tm*1*Dhr*^/J) mice were obtained from The Jackson Laboratory (Bar Harbor, ME, USA). All mice were treated in strict adherence with approved protocols (N13043NAs and N17031NAs) from the CRCHUM Institutional Committee for the Protection of Animals and the Canadian Council on Animal Care.

### Experimental Autoimmune Encephalomyelitis (EAE) Disease Induction and Scoring

Active EAE was induced as previously published (20). Briefly, 6–8 week old female mice were immunized subcutaneously with 200 μg of myelin oligodendrocyte glycoprotein 35–55 (MOG_35−55_) peptide emulsified in Complete Freund's adjuvant supplemented with 400 μg of *Mycobacterium tuberculosis* (CFA-MOG_35−55_). Two days later, mice were intraperitoneally injected with 400 ng of pertussis toxin (PTX).

For passive EAE, 6–8 week old female donor WT mice were similarly immunized with CFA-MOG_35−55_ and injected intraperitoneally with 400 ng of PTX. Eight days later, donor mice were sacrificed; lymph nodes and spleens were harvested and processed as described below. Cells were put in culture at 7 million/ml in complete RPMI [10% (v/v) of fetal bovine serum, 50 μM of β-mercaptoethanol, 1 mM of sodium pyruvate, 0.01 M of HEPES, 1X non-essential amino acids solution, 2 mM glutamine, 100 U/ml penicillin, and 100 μg/ml streptomycin] in the presence of MOG_35−55_ (20 ug/ml), recombinant mouse IL-12 (10 ng/ml, R&D Systems distributed by Cedarlane Laboratories Oakville, ON, Canada), recombinant human IL-2 (100 U/ml, Roche, Nutley, NJ) and mouse recombinant IL-15 (1 ng/ml, R&D Systems) pre-complexed (incubation 30 min at 37°C) with recombinant mouse IL-15Rα (4.67 ng/ml, R&D Systems) as published by others (21). After 72 h of culture, cells were washed, resuspended in Hank's Balanced Salt solution, filtered on 70 μm cell strainer, counted and injected intraperitoneally into naïve *Klrk1*^−/−^ and wild type (*Klrk1*^+/+^) female mice.

Passive and active EAE animals were scored according to the following scale: 0 = normal, 1 = limp tail, 2 = slow righting-reflex, 3 = paralysis of one hind limb, 3.5 = paralysis of one hind limb and weakness in second hind limb, 4 = paralysis of both hind limbs, 4.5 = paralysis of both hind limbs and weakness in forelimbs, 5 = moribund. Deeply anesthetized mice were perfused with 50 ml of saline 0.9% (w/v) prior to collect organs for RNA extraction, protein extraction, or organ processing for flow cytometry analysis.

### RNA Extraction and RT-qPCR

Collected organs were put directly into TRIzol® Reagent (Life technologies Thermo Fisher Scientic, Burlington, ON, Canada). Total RNA was extracted from spleen, liver, spinal cord, brain stem-cerebellum and forebrain according to manufacturer's instructions. RNA samples were transcribed into cDNA using Quantitect Reverse transcription kit (Qiagen, Mississauga, ON, Canada) as previously published (22). Gene expression levels were determined by quantitative real-time PCR using primers and TaqMan FAM-labeled probes from Applied Biosystems-ThermoFisher Scientific and the QuantStudio 7 Flex Real-Time PCR System. Amplification of mouse HPRT1 (Mm01545399_m1) was used as an endogenous control to assess the expression of murine NKG2D ligands: MULT1 (Mm01180648_m1), panRAE (Mm00558293_g1), H60b (Mm04243254_m1), and H60c (Mm04243526_m1).

### Protein Extraction

Spinal cord, brain stem-cerebellum and forebrain were, respectively, homogenized in 500 ul of lysis buffer [50 mM Tris pH 7.5, 1 mM EDTA, 150 mM NaCl, 1% (w/v) NP40, 1% (w/v) SDS and cocktail of protease inhibitor containing 5 μg/ml of: Leupeptin (BioShop, Burlington, ON, Canada), Pepstatin A (BioShop) and Chymostatin (Sigma-Aldrich, Oakville, ON, Canada)]. Lysates were kept 10 min at room temperature before storing at −80°C.

### Cerebrospinal Fluid (CSF) Collection

Deeply anesthetized mice were placed on the stereotaxic instrument with 135° between head and body. Sagittal incision of the skin was made aligned with the ears, then the subcutaneous tissues and muscles were separated by blunt dissection and were hold by microretractor. The capillary tube was introduced into the cisterna magna though the dura mater, laterally to the arteria dorsalis spinalis. CSF was harvested into capillary tube, collected into Eppendorf tube and immediately frozen on dry ice. Samples were stored at −80°C.

### Western Blot

Proteins isolated from organs were electrophoresed under reducing conditions and transferred onto PVDF membrane as previously published (23). CSF samples were run on a stain free gel (Bio-Rad Laboratories, Mississauga, ON, Canada) containing trihalo compounds reacting with tryptophan residues and used to revel total proteins on gel before transfer. MULT1 (rat monoclonal antibody clone 1D6 provided by Dr. S. Jonjic, University of Rijeka, Rijeka, Croatia) and Rae-1 (polyclonal goat anti-Rae-1 pan specific R & D Systems), were quantified by chemiluminescence relative to total protein as published (24), actin (monoclonal antibody clone C4, MP Biomedicals, Fisher Scientific) or albumin (rabbit anti-albumin, Novus Biologicals, distributed by Cedarlane Laboratories) using the Chemidoc MP imaging system (Bio-Rad Laboratories).

### Immune Cell Isolation From CNS and Lymphoid Organs

Brain and spinal cord were processed from individual mice as previously published (20). Briefly, minced CNS was digested 15 min with collagenase D and DNase I at 37°C prior to being mashed. Myelin was removed using Percoll. Harvested spleens and lymph nodes were put in complete RPMI and then mashed through cell strainer (70 μm) to obtain single cell suspension. Red blood cells from spleens were lysed using 0.83% (w/v) of ammonium chloride for 4 min at room temperature.

### Culture of Splenocytes

Splenocytes from *Klrk1*^+/+^ or *Klrk1*^−/−^ mice were put in culture in complete RPMI in 48 well plates pre-coated with anti-CD3 antibody (2 ug/ml in 100 mM NaHCO3 overnight at 4°C, BD Biosciences, Mississauga, ON, Canada), anti-CD28 antibody (1 ug/ml BD Biosciences), recombinant human IL-2 (100 U/ml), and the complex recombinant mouse IL-15/IL-15Rα (described above). On day 5, recombinant murine MULT1 protein (10 μg/ml, R&D Systems) was added or not to splenocytes. On day 7, cells were activated for the detection of intracellular mediators and analyzed by flow cytometry as described below.

### Flow Cytometry

Flow cytometry staining was performed as previously published (22, 25, 26). Briefly, cells were incubated 15 min in blocking solution containing normal mouse immunoglobulins and rat anti-mouse CD16/CD32 prior to be incubated in the presence of fluorochrome-conjugated antibodies. Antibodies targeted CD45.1 (Biolegend, San Diego, CA, USA, clone A20), CD45.2 (BioLegend, distributed by Cedarlane Laboratories, clone 104), CD11b (BD Biosciences, clone M1/70), CD3 (BD Biosciences, clone 145-2C11), CD4 (BD Biosciences, clone RM4-5), CD8 (BD Biosciences, clone 53-6.7), and NKG2D (eBioscience or R&D systems, respectively, clone CX5 or 191004). LIVE/DEAD Fixable Aqua Dead Cell Stain was used to exclude dead cells. To trace proliferation of MOG-specific T cells, leukocytes from MOG-immunized donor mice were labeled with CFSE as previously done (22, 25) prior to being activated *in vitro* for 72 h. For cytokine detection, cells were stimulated 5 h with phorbol 12-myristate 13-acetate (20 ng/ml, Sigma-Aldrich) and ionomycin (1 ug/ml, Sigma-Aldrich) in the presence of brefeldin A (5 ug/ml, Sigma-Aldrich) and monensin sodium (1 μM Sigma-Adrich). Intracellular staining was accomplished as previously published (25). Antibodies targeted interferon-γ (IFNγ, BD Biosciences clone MP6-XT22), granulocyte-macrophage colony-stimulating factor (GM-CSF, BD Biosciences, clone MP1-22E9), interleukin-17 (IL-17, BD Biosciences, cloneTC11-18H10) and granzyme B (eBioscience ThermoFisher Scientific, clone 16G6). Appropriate isotype controls were used in all steps. Staining specificity was confirmed using fluorescence minus one (FMO: all antibodies minus one). The median fluorescence intensity (MFI) was calculated by subtracting the fluorescence of the isotype from that of the stain. Cell numbers were quantified using either cell counting prior to cytometry staining or beads added to samples prior to sample acquisition as previously described (20).

### Immunohistochemistry

Deeply anesthetized mice were perfused with 30 ml of saline 0.9% (w/v) and then with 50 ml of paraformaldhehyde 4% (w/v in PBS). Spinal cord was collected and soaked into 4% paraformaldehyde for 1 day prior to being transferred into sucrose 30% (w/v) for 2 days and then put into OCT for freezing at −80°C. Nine micron sections were stained for FluoroMyelin™ Green fluorescent myelin stain (Thermofisher Scientific) and 2-(4-Amidinophenyl)-6-indolecarbamidine dihydrochloride, 4′,6-Diamidino-2-phenylindole dihydrochloride (DAPI) (Sigma-Aldrich) for nucleus detection according to manufacturers' instructions. Slides were observed using a SP5 Leica confocal microscope and confocal images acquired sequentially in different channels using LASAF software and overlay using Adobe Photoshop software.

### Statistical Analysis

Data analysis was performed using Prism 7.0 software (GraphPad, La Jolla, CA, USA). Statistical tests used are indicated in figure legends. Values were considered statistically significant when probability (P) values were equal or below 0.05 (^*^), 0.01 (^**^), or 0.001 (^***^).

## Results

### NKG2D has a Modest Impact on Active EAE

**Figure 1:**
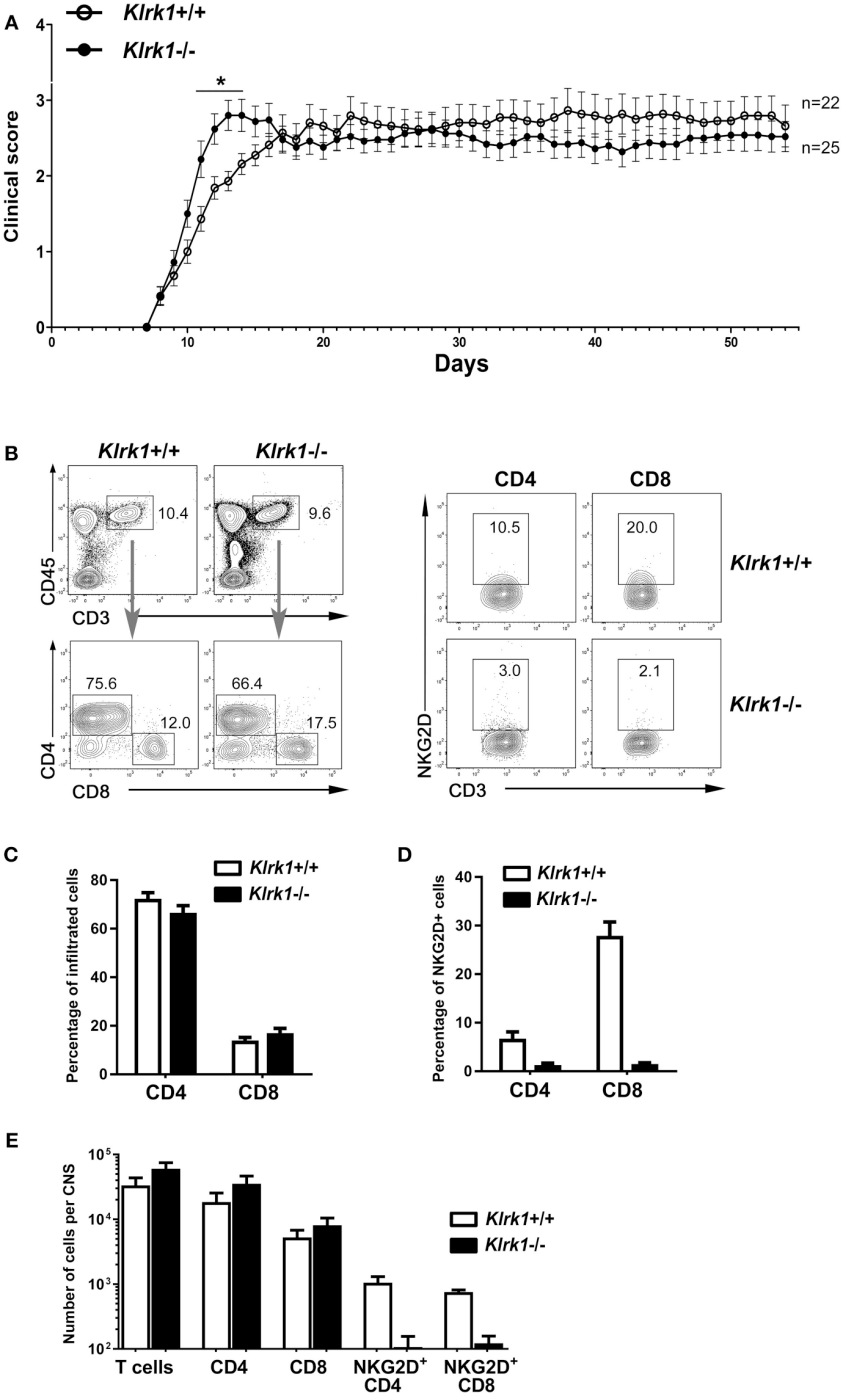
Active EAE disease course is similar in the absence of NKG2D. Active EAE was induced in *Klrk1*^−/−^ (black circles) and wild type (*Klrk1*^+/+^, open circles) mice. Clinical score was assessed over 55 days or CNS infiltrating T lymphocytes characterized by flow cytometry. **(A)** Clinical score of *Klrk1*^+/+^ (*n* = 22) and *Klrk1*^−/−^ mice (*n* = 25) presented as mean ± SEM. 2way ANOVA *Klrk1*^−/−^ vs. *Klrk1*^+/+^ day 11 to 14 ^*^p < 0.05. **(B)** Representative contour plots show gating for T cells (CD45^hi^CD3^+^) and then CD4 and CD8 as well as the detection of NKG2D on CD4 and CD8 T cell subsets from CNS cells of *Klrk1*^−/−^ and *Klrk1*^+/+^ mice. **(C)** Percentage of CNS infiltrated CD4 and CD8 T lymphocytes within CD3 T cells. Mean ± SEM *n* = 3. **(D)** Percentage of CNS-infiltrated CD4 and CD8 T lymphocytes expressing NKG2D. Mean ± SEM *n* = 3. **(E)** Number of cells per CNS (spinal cord and brain pooled from each individual mouse) for T cells (CD45^hi^CD3^+^), CD4 and CD8 T cells as well as NKG2D^+^CD4 T cells and NKG2D^+^CD8 T cells. Mean ± SEM *n* = 3.

To assess the impact of NKG2D on the development of EAE, we applied our standard protocol on mice lacking NKG2D (*Klrk1*^−/−^) and wild type (*Klrk1*^+/+^) counterparts. We observed a modest and transient (day 11 to 14) enhanced peak of disease in *Klrk1*^−/−^ compared to wild type mice when large groups of animals were analyzed (*n* = 22–25) (Figure 1A). We observed similar proportions of infiltrating CD4 (67–72% of total CD3 T lymphocytes) and CD8 (14–17% of total CD3 T lymphocytes) T cells in the CNS of both groups (Figures 1B,C). Within these CNS-infiltrated T lymphocytes, only a very small proportion of CD4 T lymphocytes expressed NKG2D (less than 8%) whereas 28% of CD8 T lymphocytes carried detectable levels of this receptor in *Klrk1*^+/+^ mice (Figure 1D). As expected, a negligible proportion of T lymphocytes expressed NKG2D in the *Klrk1*^−/−^ mice (Figures 1B,D). The total number of T cells, CD4 and CD8 T cells that infiltrated the CNS during EAE were similar in both genotypes (Figure 1E). Similar to what others published (19), NKG2D had a minimal impact on the neurobehavioral score of EAE mice. Our results show that during EAE, amongst the CNS-infiltrated T lymphocytes, a greater proportion of CD8 than CD4 T lymphocytes carried NKG2D but as CD4 T cells were more abundant, NKG2D-expressing CD4 and CD8 T lymphocytes were present in similar number per CNS in wild type mice (Figure 1E).

### EAE Induction Modestly Alters the Expression of Rae-1 and H60 Ligands

**Figure 2:**
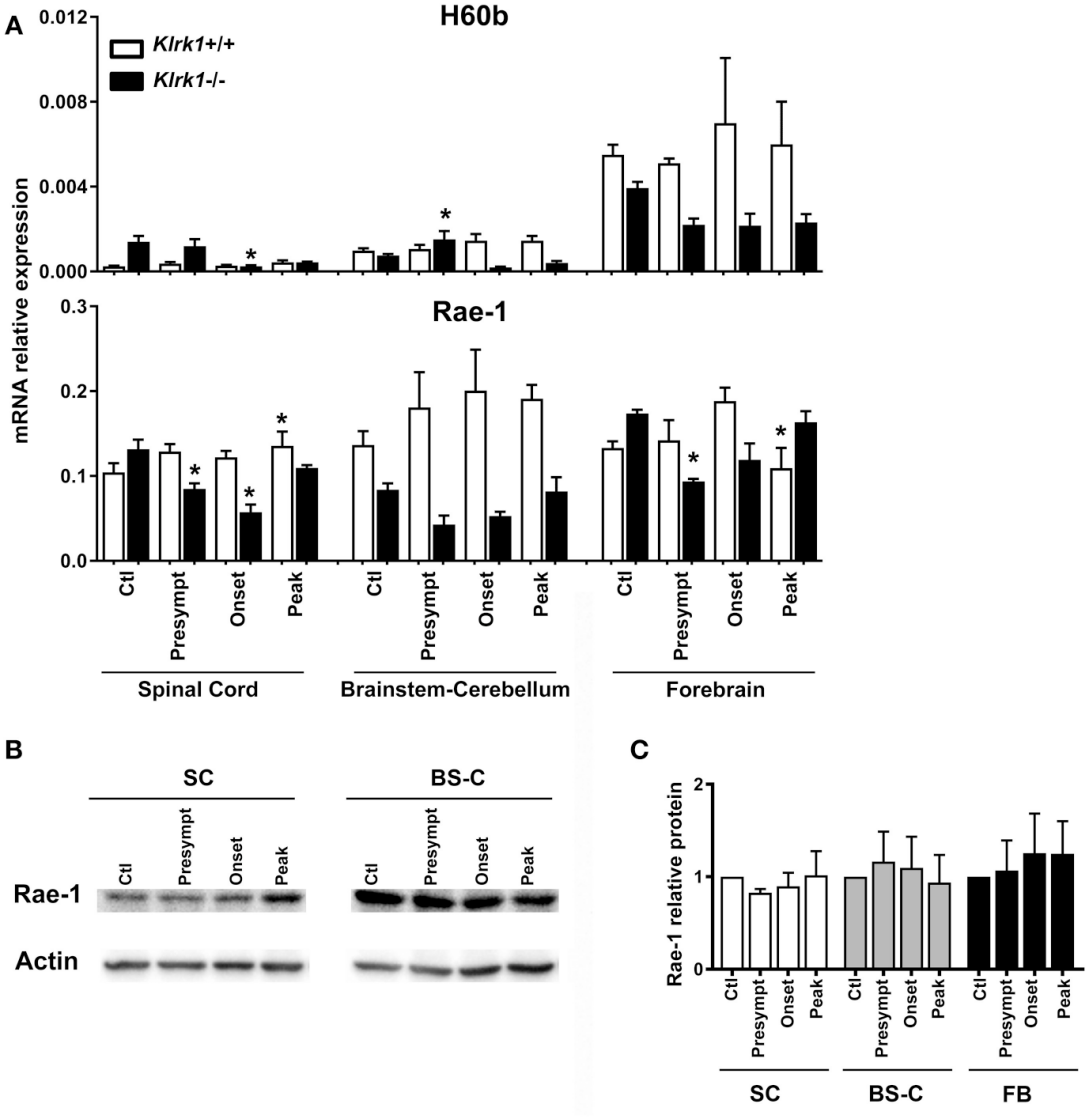
Variations in CNS expression of Rae-1 only at the mRNA level. **(A)** Relative mRNA expression of H60b and Rae-1 in CNS areas [spinal cord (SC), brainstem-cerebellum (Bs-C) and forebrain (Fb)] from *Klrk1*^−/−^ (black bars) and wild type (*Klrk1*^+/+^; white bars) mice either injected with CFA-PBS as control (Ctl) or subjected to active EAE and sacrificed at pre-symptomatic (presympt), onset or peak stage of disease. Kruskal-Wallis test and Dunn's test analysis within the same genotype group. Statistical differences for H60b in *Klrk1*^−/−^ group: spinal cord onset vs. control or presymptomatic; brainstem-cerebellum presymptomatic vs. onset or peak. Statistical differences for Rae-1 expression in *Klrk1*^+/+^ group: spinal cord: peak vs. ctl; forebrain onset vs. peak. Statistical differences for Rae-1 expression in *Klrk1*^−/−^ group: spinal cord control vs. presymptomatic or onset; for forebrain presymptomatic vs. control or peak. ^*^p < 0.05. **(B,C)** Western blot analysis of Rae-1 expression in the spinal cord (SC, brainstem-cerebellum (Bs-C) and forebrain (Fb) from wild type mice (*Klrk1*^+/+^) either injected with CFA-PBS as control (Ctl) or subjected to active EAE and sacrificed at pre-symptomatic (presympt), onset or peak stage of disease. **(B)** One representative Western blot and **(C)** Rae-1 relative expression to actin, ctl for each organ defined as 1. Mean ± SEM *n* = 5.

As each NKG2DL can be induced in response to different triggers and exhibits distinct biological functions, we assessed the expression of the specific ligands (H60 (b-c), Rae1 (α-ε) and MULT1) known to be transcribed in C57BL/6 mice (9, 27–29), during the development of EAE. To investigate whether the presence of NKG2D-expressing immune effectors affects the ligands' expression, we quantified the relative mRNA levels of NKG2DL in *Klrk1*^−/−^ and wild type (*Klrk1*^+/+^) mice at the different EAE stages: pre-symptomatic, onset and peak stages. We analyzed three CNS areas: spinal cord, brain stem/cerebellum and forebrain and observed that the mRNA levels of H60b did not significantly vary in the wild type mice (Figure 2A). We detected a slight but significant decrease in the spinal cord of *Klrk1*^−/−^ mice compared to controls at disease onset and a slight increase in the brainstem-cerebellum (Figure 2A). H60c mRNA was not detected in all tested organs (CNS, spleen, etc.) except the eye (data not shown). Using a pan-Rae-1 qRT-PCR assay to detect all forms of Rae-1, we observed a small but significant increase at the peak of disease in wild type mice in the spinal cord compared to controls as previously reported by others (30). However, Rae-1 mRNA levels were decreased in the spinal cord of *Klrk1*^−/−^ mice at the presymptomatic and onset stages as well as in the forebrain at the presymptomatic stage (Figure 2A). Notably, the levels in *Klrk1*^−/−^ mice were significantly lower than in *Klrk1*^+/+^ at the same disease stage for the presymptomatic and onset in the spinal cord and the brainstem-cerebellum (Figure 2A). We also assessed the protein expression of Rae-1 in these CNS areas during the development of EAE. The relative amounts of Rae-1 did not vary between control mice (CFA without MOG) and EAE animals in all three CNS areas tested (spinal cord, brainstem/cerebellum, forebrain) (Figures 2B,C).

### Enhanced Expression of MULT1 in the CNS of EAE Mice

**Figure 3:**
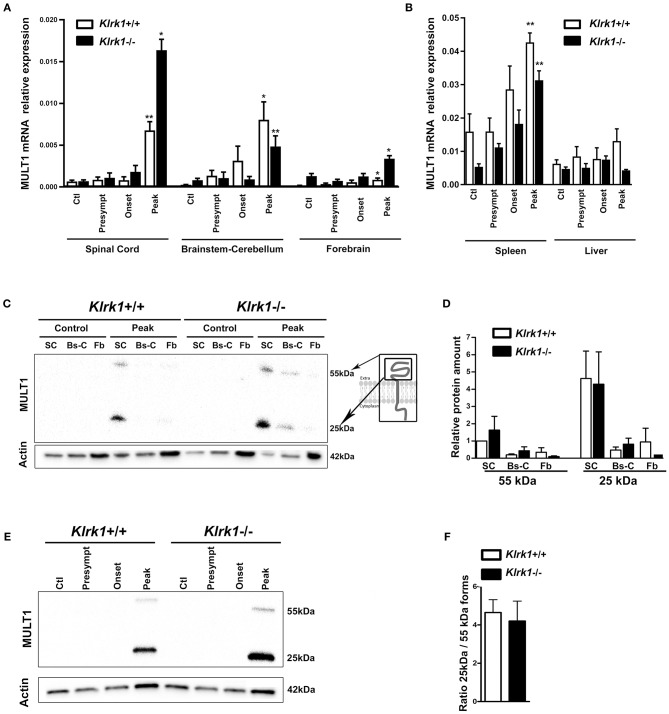
Upregulation of MULT1 at the mRNA and protein levels in the CNS during EAE. Quantification of MULT1 expression in organs from *Klrk1*^−/−^ (black bars) and wild type (*Klrk1*^+/+^; white bars) mice either injected with CFA-PBS as control (Ctl) or subjected to active EAE and sacrificed at pre-symptomatic (presympt), onset or peak stage of disease. **(A)** MULT1 relative mRNA expression in the spinal cord (SC), brainstem-cerebellum (Bs-C) and forebrain (Fb). Mean ± SEM *n* = 4–8. Kruskal-Wallis test and Dunn's test analysis within the same genotype group; statistical differences in *Klrk1*^+/+^ group: spinal cord and brainstem-cerebellum: peak vs. ctl, presympt, or onset; forebrain peak vs. control. Statistical differences within *Klrk1*^−/−^ group: spinal cord peak vs. control, presymptomatic or onset stage; brainstem-cerebellum peak vs. presymptomatic; forebrain peak vs. presymptomatic. ^*^p < 0.05; ^*^^*^p < 0.01. **(B)** MULT1 relative mRNA expression in spleen and liver. Kruskal-Wallis test and Dunn's test analysis within the same genotype group; statistical differences in spleen for both *Klrk1*^+/+^ and *Klrk1*^−/−^ groups: peak vs. ctl. ^*^^*^p < 0.01. **(C–F)** Western blot analysis of MULT1 and actin levels in different CNS areas from *Klrk1*^−/−^ and *Klrk1*^+/+^ mice at different disease stages. **(C)** One representative western blot of spinal cord (SC), brainstem-cerebellum (Bs-C) and forebrain (Fb) from control mice and mice at EAE peak; cartoon illustrating full and cleaved forms of MULT1. **(D)** Quantification of 55 kDa and 25 kDa forms as relative expression compared to actin at disease peak in *Klrk1*^−/−^ (black bars) and *Klrk1*^+/+^ (white bars) mice; levels in *Klrk1*^+/+^ spinal cord at disease peak defined as 1. Mean ± SEM *n* = 4–6. **(E)** One representative western blot of spinal cord from *Klrk1*^−/−^ and *Klrk1*^+/+^ mice treated as control or EAE at different disease stages. **(F)** Ratio of the 25kDa on 55 kDa forms in the spinal cord at peak of disease for both genotypes. *Klrk1*^−/−^ (black bars) and *Klrk1*^+/+^ (white bars). Mean ± SEM *n* = 4–6.

In contrast, MULT1 mRNA levels were significantly enhanced in the spinal cord of both *Klrk1*^+/+^ and *Klrk1*^−/−^ groups at the peak of EAE compared to controls, presymptomatic and onset time points (Figure 3A). Upregulated MULT1 mRNA levels during EAE were previously reported by others (30). Moreover, MULT1 mRNA levels were greater in *Klrk1*^−/−^ mice compared to wild type counterparts (Figure 3A) without reaching statistical significance. These observations suggest that in the presence of NKG2D-expressing immune effectors, MULT1-expressing cells may down-regulate such expression or may be destroyed. In the brainstem-cerebellum and forebrain, we also detected significantly augmented MULT1 mRNA levels at the peak of EAE compared with other disease stages in both *Klrk1*^+/+^ or *Klrk1*^−/−^ mice (Figure 3A). We observed a small increase of MULT1 mRNA levels in the spleen of *Klrk1*^−/−^ and *Klrk1*^+/+^ mice at disease peak and no variation in the liver (Figure 3B). These results demonstrate that during active EAE, MULT1 expression is significantly upregulated (over 900% increase in spinal cord) in the CNS.

To determine whether MULT1 expression is also increased at the protein level, we performed western blot analysis on the same CNS areas. We observed two bands representing the full length (50–55 kDa) and the putative cleaved extracellular domain (25 kDa) of MULT1 at the peak of disease, which was absent in controls mice (Figure 3C). The quantification of MULT1 relative to actin showed that the spinal cord contained greater levels of both MULT1 forms (55 and 25 kDa) compared to brainstem/cerebellum and forebrain areas in *Klrk1*^+/+^ as well as *Klrk1*^−/−^ mice at disease peak (Figure 3D). The full and cleaved forms of MULT1 were detected in the spinal cord at the onset of EAE only in a subset of mice (4 out of 12 mice). Notably, greater amounts of the 25 kDa band relative to the 55 kDa band at disease peak were observed in both *Klrk1*^+/+^ and *Klrk1*^−/−^ mice (Figures 3E,F). Our results strongly suggest that MULT1 is the NKG2DL with the greatest enhanced mRNA and protein expression levels at the peak of EAE in the CNS, especially in the spinal cord, regardless of the presence of NKG2D-expressing immune cells.

### Elevated Expression of Soluble MULT1 in the CSF of EAE Mice

**Figure 4:**
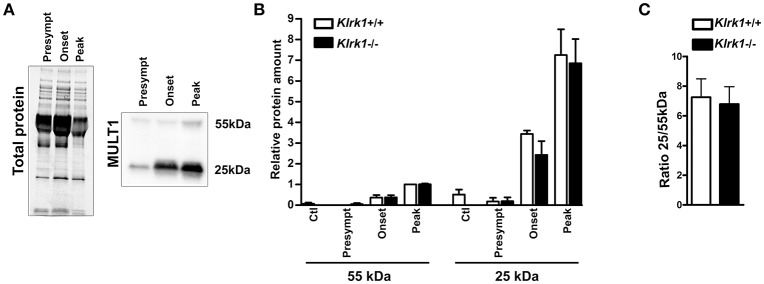
Increased levels of soluble MULT1 in the CSF during EAE. Quantification of soluble MULT1 in CSF from *Klrk1*^−/−^ (black bars) and wild type counterparts (*Klrk1*^+/+^; white bars) either injected with CFA-PBS as control (Ctl) or subjected to active EAE and sacrificed at pre-symptomatic (presympt), onset or peak stage of the disease. **(A)** One representative western blot for the detection of total protein and MULT1. **(B)** Quantification of 55 kDa and 25 kDa forms as relative expression compared to total protein; levels in *Klrk1*^+/+^ at disease peak defined as 1. Mean ± SEM *n* = 3–4. **(C)** Ratio of the 25 kDa/55 kDa forms at disease peak. Mean ± SEM *n* = 4.

To establish whether soluble MULT1 diffuses in the CNS, we performed western blots on CSF collected from control or EAE mice at different stages (pre-symptomatic, onset, peak) in *Klrk1*^+/+^ and *Klrk1*^−/−^ mice (Figure 4A). We observed that both forms (25 and 55 kDa) of MULT1 were detectable in the CSF and those levels were greater at disease peak compared to other EAE stages (Figure 4B). Notably, the 25kDa form was 7-fold more abundant than the 55 kDa form in the CSF (Figure 4C) compared to 4.5-fold increased expression in the spinal cord protein homogenates at the same disease stage (Figure 3F).

### Soluble MULT1 Released During EAE Enhances T Cell Responses

**Figure 5:**
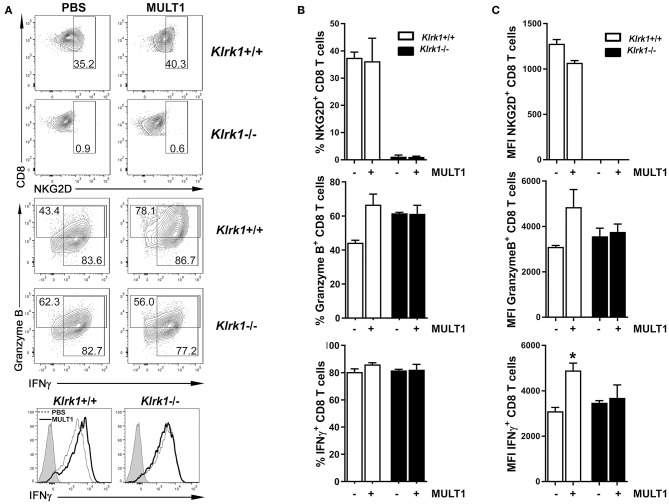
Soluble MULT1 augments effector functions of CD8 T lymphocytes. Splenocytes from naïve *Klrk1*^−/−^ (black bars) and wild type (*Klrk1*^+/+^ white bars) mice were activated with anti-CD3, anti-CD28, IL-2, and IL-15 for 5 days and then exposed or not to soluble recombinant MULT1 for an additional 2 day culture. Cells were subsequently shortly activated and analyzed by flow cytometry for the expression of CD3, CD8, NKG2D, granzyme B, and IFNγ. **(A)** Representative contour plots are illustrated for the detection of NKG2D or Granzyme B and IFNγ on CD3^+^CD8^+^ T cell-gated cells from *Klrk1*^−/−^ and *Klrk1*^+/+^ mice. Histograms showing IFNγ on CD3^+^CD8^+^ T cell-gated cells from *Klrk1*^−/−^ and *Klrk1*^+/+^ mice according to treatment: PBS (dotted line) or MULT1 (filled line); gray filled represents isotype control. **(B)** Percentage of CD8 T lymphocytes expressing NKG2D, Granzyme B or IFNγ. Mean ± SEM *n* = 3 individual mice of one representative experiment. **(C)** MFI intensity for CD8 T lymphocytes expressing NKG2D, Granzyme B or IFNγ. Mean ± SEM *n* = 3 individual mice of one representative experiment. Statistical differences within *Klrk1*^+/+^ group without vs. with MULT1 ^*^p < 0.05.

We next sought to determine whether soluble MULT1 could impact activated T lymphocytes, which are abundantly present in the CNS of EAE mice. Splenocytes from naïve *Klrk1*^+/+^ and *Klrk1*^−/−^ mice were activated *in vitro* with anti-CD3+anti-CD28+IL-2+IL-15 to enhance NKG2D expression on CD8 T lymphocytes to similar levels observed on CNS-infiltrating CD8 T cells. After 5 days, recombinant mouse MULT1 or PBS was added to these activated cultures and 2 days later, cells were evaluated for their production of mediators by flow cytometry. Recombinant MULT1 was obtained from the same commercial source than what has been used by the Raulet's group to perform *in vitro* experiments with activated murine immune cells (10). Our *in vitro* culture conditions gave rise to a similar proportion (37%) of activated *Klrk1*^+/+^ CD8 T lymphocytes expressing NKG2D (Figures 5A,B) as we observed on CNS-infiltrating CD8 T lymphocytes in EAE mice (Figure 1C, 28%). As expected, NKG2D expression was negligible on *Klrk1*^−/−^ cells. Notably, the addition of soluble MULT1 did not alter the percentage of cells expressing NKG2D; but slightly reduced NKG2D expression levels per cell in *Klrk1*^+/+^ CD8 T cells [Figure 5C; MFI for NKG2D on *Klrk1*^+/+^ CD8 T cells exposed to PBS (1279) vs. MULT1 (1068)]. The addition of MULT1 increased the percentage of granzyme B-expressing CD8 T lymphocytes (Figure 5B; % granzyme-expressing *Klrk1*^+/+^ CD8 T cells exposed to PBS (44%) vs. MULT1 (67%) *p* = 0.09); these cells expressed greater levels of this lytic enzyme compared to cells not treated with MULT1 (Figure 5C; granzyme B-MFI in *Klrk1*^+/+^ CD8 T cells exposed to PBS (3094) vs. MULT1 (4854) *p* = 0.1). MULT1 did not alter the percentage of IFNγ producing *Klrk1*^+/+^ CD8 T lymphocytes (80–86%) but significantly increased their cellular expression level (Figures 5A,C; IFNγ-MFI on *Klrk1*^+/+^ CD8 T cells exposed to PBS (3095) vs. MULT1 (4901) ^*^p = 0.0133). The enhancing impact of MULT1 on CD8 T cell effector functions was dependent on the presence of NKG2D as this soluble ligand did not alter the production of granzyme B or IFNγ by *Klrk1*^−/−^ CD8 T lymphocytes as similar percentage of cells and MFI were observed for these cells in the PBS and MULT1 conditions (Figure 5). Our results suggest a novel activating role of soluble MULT1 on CD8 T lymphocytes.

### NKG2D Significantly Augments Passive EAE Severity

**Figure 6:**
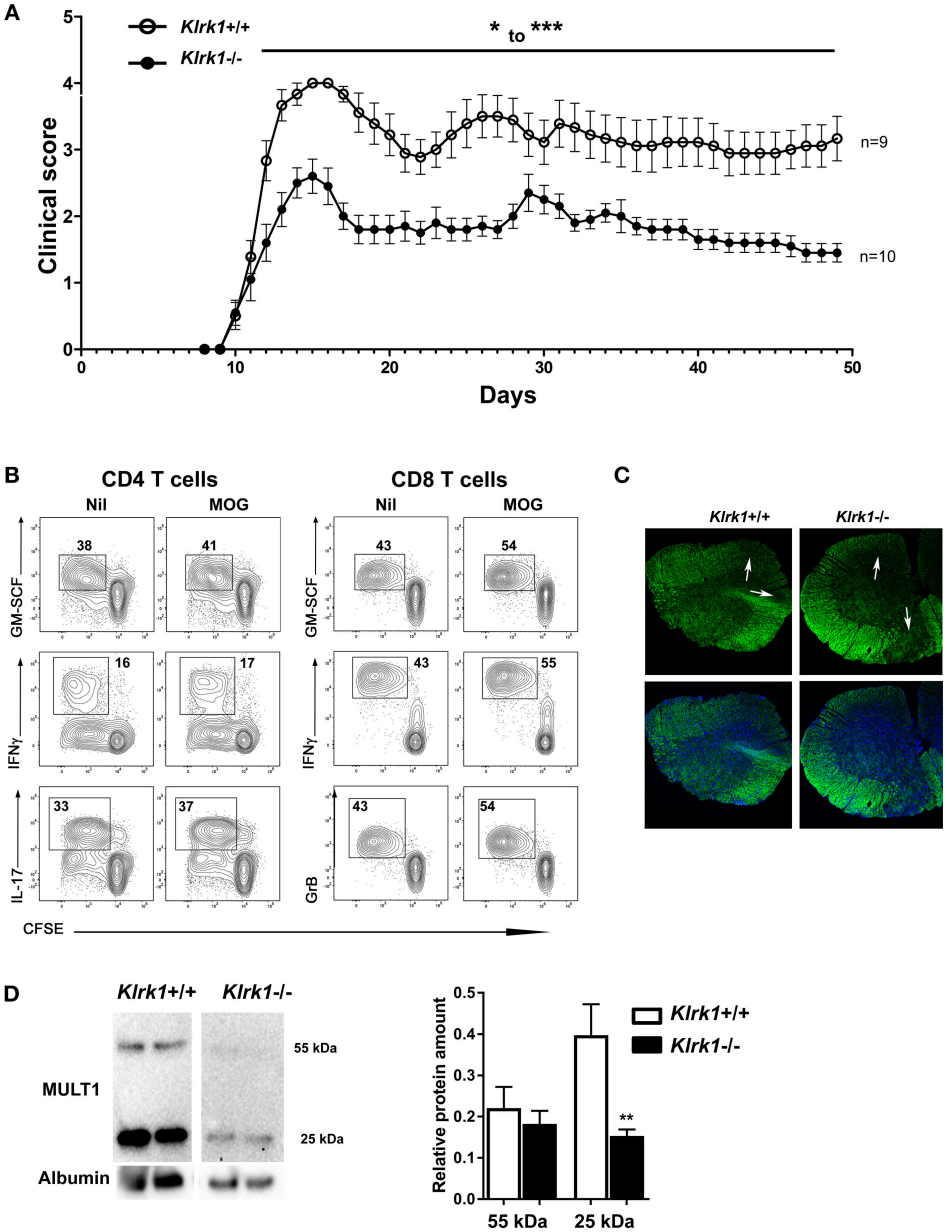
Passive EAE is less severe in *Klrk1*^−/−^ than in wild type recipients. Leukocytes from MOG-immunized C57BL/6 were reactivated *in vitro* and then adoptively transferred to *Klrk1*^−/−^ (black) and wild type (*Klrk1*^+/+^; white) recipients. **(A)** Mice were followed for clinical score. Mean ± SEM, *n* = 9–10. 2way ANOVA *Klrk1*^−/−^ vs. *Klrk1*^+/+^ day 22–23 ^*^p < 0.05 for all other days from day 12 to the end: ^*^^*^^*^p < 0.001. **(B)** Flow cytometry analysis of *ex vivo* expanded T lymphocytes from MOG-immunized donor mice. Lymph node cells were collected, labeled with CFSE, and put in culture as described in materials and methods in the absence or presence of MOG_35−55_ for 72 h. PMA, ionomycin, brefeldin A and monensin were added for 4 h prior to flow cytometry staining and analysis for intracellular mediators: GM-CSF, IFNγ, IL-17 and Granzyme B (GrB) as indicated. Contour plots illustrate gated events on CD4 or CD8 T cells expanded *in vitro* in the absence (Nil) or presence of MOG. **(C)** Representative detection of demyelination using fluoromyelin and DAPI in spinal cord sections from *Klrk1*^−/−^ and *Klrk1*^+/+^ mice. White arrows indicate zones of myelin loss. **(D)** Western blot detection of MULT1 and albumin in CSF from *Klrk1*^−/−^ and *Klrk1*^+/+^ mice 50 days after the adoptive transfer of activated lymphocytes. One representative western blot is illustrated and quantification of 55 kDa and 25 kDa forms as relative expression compared to albumin Mean ± SEM *n* = 7. Statistical differences for relative abundance of MULT1 25kDa form *Klrk1*^+/+^ vs. *Klrk1*^−/−^^*^^*^p < 0.01.

Active EAE is strongly mediated by CD4 T lymphocytes but only a small proportion of these lymphocytes can express NKG2D upon activation. In contrast, a great proportion of activated murine CD8 T cells acquire NKG2D. To investigate the contribution of this receptor, we optimized a passive EAE model in which the transferred T cells include a similar proportion of CD8 and CD4 T cells. Lymphocytes from MOG-immunized donor mice were activated *in vitro* in the presence of IL-2, IL-12, and IL-15 prior to their transfer into naïve recipients. The addition of this cytokine cocktail led to the expansion of similar proportions of CD4 and CD8 T cells (data not shown). Activated cells were transferred into naive *Klrk1*^+/+^ and *Klrk1*^−/−^ mice and clinical score recorded for 2 months (Figure 6A). Disease onset occurred similarly around day 10 post-transfer in both mouse groups (Figure 6A). However, *Klrk1*^+/+^ recipients achieved a significantly more elevated clinical score than the *Klrk1*^−/−^ recipients (Figure 6A). The dampened disease in *Klrk1*^−/−^ recipients was maintained for the whole observation window. As these mice were injected in parallel with the same activated lymphocytes, the differences we observed could not be due to the transferred cells. Our results suggest that in passive EAE, endogenous immune cells contribute to disease pathobiology and that such impact depends on the endogenous expression of NKG2D.

We evaluated whether the injected cells contain MOG-specific T cells. Lymph node leukocytes from MOG-immunized donor mice were labeled with CFSE and activated *in vitro* following our usual protocol in the presence of MOG. Control cells were cultured in the same conditions but in the absence of the myelin peptide. We observed that our activation protocol efficiently induced proliferation as assessed by CFSE dilution (Figure 6B). Moreover, the proportion of CD4 and CD8 T lymphocytes that divided during the *in vitro* expansion and could produce key immune effectors (GM-CSF, IFNγ, IL-17 for CD4; GM-CSF, IFNγ, and Granzyme B for CD8) was greater in the presence of MOG (Figure 6B). We suggest that *in vitro* MOG addition had a more modest impact on CD4 than CD8 T lymphocytes due to the greater efficiency of antigen presenting cells activated upon immunization at activating CD4 than CD8 T lymphocytes. These results support the idea that at least a subset of transferred T lymphocytes was MOG-specific. In addition, the passive transfer of MOG-activated lymphocytes caused demyelination in recipient mice. Indeed, fluoromyelin staining revealed zones of demyelination (white arrows Figure 6C) in both *Klrk1*^+/+^ and *Klrk1*^−/−^ mice. Finally, we also detected soluble MULT1 in the CSF of passive EAE mice supporting the notion that this NKG2DL is also elevated in this form of disease (Figure 6D). Notably, the relative amount of the 25kDa form of MULT1 was significantly more elevated in CSF from *Klrk1*^+/+^ compared to *Klrk1*^−/−^ (Figure 6D).

### CNS-Infiltrated Endogenous CD8 T Cells Exhibit Effector Functions

**Figure 7:**
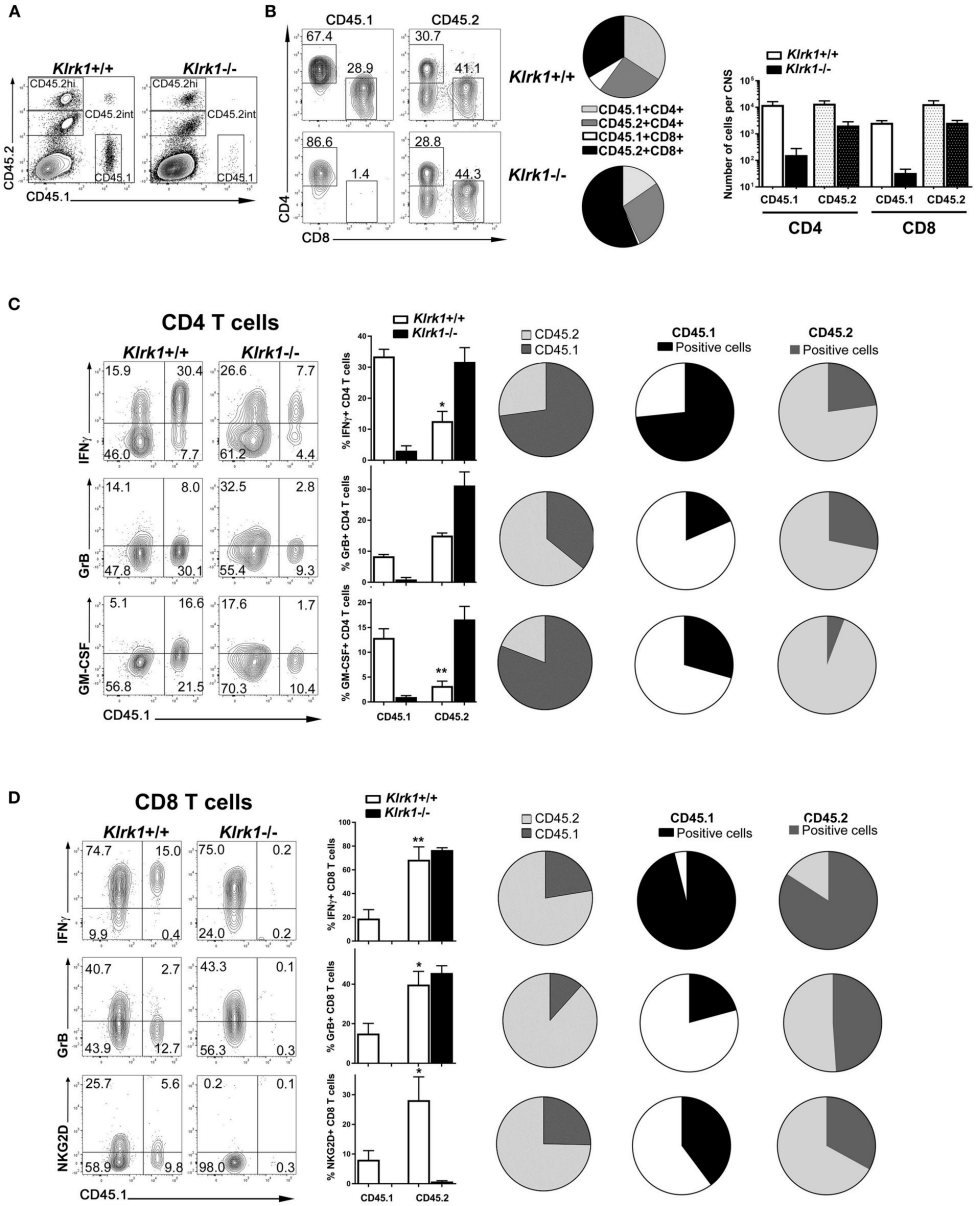
CNS-infiltrated endogenous CD8 lympocytes exhibit effector functions. Leukocytes from MOG-immunized B6-CD45.1 were reactivated *in vitro* and then adoptively transferred into CD45.2-expressing *Klrk1*^−/−^ and *Klrk1*^+/+^ mice. Recipients were sacrificed on day 25 and organs collected for flow cytometry analysis. Cells from donor mice were CD45.1 whereas cells from recipients express the standard C57BL/6 CD45.2 marker. Flow data representative of 3–4 mice per group. **(A)** Representative contour plots of CNS cells in *Klrk1*^−/−^ and *Klrk1*^+/+^ recipient mice showing CD45.1^+^ cells (donor cells) or CD45.2^+^ cells (endogenous cells) amongst all single and living cells. **(B)** Representative contour plots showing CD45.1^+^ or CD45.2^+^ gated cells for CD4 and CD8 T cell detection in *Klrk1*^−/−^ and *Klrk1*^+/+^ recipients. Pie charts illustrate the proportion of CNS-infiltrating CD3 T cells that were either CD45.1^+^CD4^+^, CD45.2^+^CD4^+^, CD45.1^+^CD8^+^, and CD45.2^+^CD8^+^ in *Klrk1*^+/+^ and *Klrk1*^−/−^ mice. Number of cells per CNS (spinal cord and brain pooled from each individual mouse) for CD4 and CD8 T cells that expressed either CD45.1 or CD45.2. Mean + SEM *n* = 3. **(C,D)** Representative contour plots of CNS cells gated either on CD4 **(C)** or CD8 **(D)** T cells in *Klrk1*^−/−^ and *Klrk1*^+/+^ recipients showing donor cells (CD45.1^+^) or endogenous cells (CD45.1 negative) producing IFNγ, granzyme B (GrB) or GM-CSF or expressing NKG2D. Bar graphs illustrate mean ± SEM *n* = 3–4 individual mice of one representative experiment. Statistical differences for percentage of IFNγ producing CD4 T cells in *Klrk1*^+/+^: CD45.1 in vs. CD45.2 ^*^p < 0.05; percentage of GM-CSF producing CD4 T cells in *Klrk1*^+/+^: CD45.1 in vs. CD45.2 ^*^^*^p < 0.01; for percentage of IFNγ producing CD8 T cells in *Klrk1*^+/+^: CD45.1 in vs. CD45.2 ^*^^*^p < 0.01; percentage of granzyme B producing CD8 T cells in *Klrk1*^+/+^: CD45.1 in vs. CD45.2 ^*^p < 0.05; percentage of NKG2D-expressing CD8 T cells in *Klrk1*^+/+^: CD45.1 in vs. CD45.2 ^*^p < 0.05. All pies show proportion of T cells in *Klrk1*^+/+^ recipients. Pies in first column illustrate percentage of T cells producing IFNγ, granzyme B (GrB) or GM-CSF or expressing NKG2D according to the expression of CD45.2 (light gray) or CD45.1 (dark gray). Pies in second (CD45.1) and third (CD45.2) columns illustrate percentage of cells producing IFNγ, granzyme B (GrB) or GM-CSF or expressing.NKG2D within these two subsets.

To investigate the contribution of endogenous vs. transferred T cells, we used C57BL/6 mice expressing the pan leukocyte marker CD45.1 as donor mice for the transfer into the CD45.2-expressing *Klrk1*^+/+^ and *Klrk1*^−/−^ recipients. Mice were sacrificed 25 days post-transfer and CNS cells were analyzed by flow cytometry. We could easily discriminate donor (CD45.1^+^) from recipient immune cells (CD45.2^+^) (Figure 7A). CD45^int^ CD11b^+^ microglia all expressed the endogenous CD45.2 allele (data no shown). We compared CD45.2^hi+^ immune cells to the CD45.1^+^ cells in *Klrk1*^+/+^ and *Klrk1*^−/−^ recipients. Most transferred cells (CD45.1^+^) detected in the CNS were either CD4 or CD8 T cells (Figure 7B). Despite the injection of similar numbers of CD45.1^+^ CD4 and CD8 T cells, donor CD8 T cells represented a very small proportion of CNS-infiltrated T cells, especially in *Klrk1*^−/−^ mice, while donor CD4 T cells were well represented. In contrast, amongst the endogenous immune cells present within the CNS, a greater proportion were CD8 T cells (41 % in *Klrk1*^+/+^ and 44% in *Klrk1*^−/−^) compared to CD4 T cells (30.7% and 28.8%) (Figure 7B). The spleens of all animals contained very low proportion of CD45.1^+^ (donor) T cells (around 1%) suggesting that the transferred cells either died or migrated to other sites such as the CNS at the time point tested (over 20 days after transfer). Overall, less transferred T cells were detected in the CNS of *Klrk1*^−/−^ recipients than wild type counterparts correlating with the reduced disease severity in *Klrk1*^−/−^ recipients (Figure 7B).

To characterize the properties of CNS-infiltrated T cells, we assessed the production of key immune effector molecules: IFNγ, GM-CSF and granzyme B in CD45.1^+^ or CD45.1-negative (CD45.2^+^) CD4 and CD8 T cells in both *Klrk1*^+/+^ and *Klrk1*^−/−^ recipients. Production of IFNγ by CNS-infiltrated CD4 and CD8 T cells was easily detected in both mouse groups. Notably, a significantly lower proportion of endogenous (CD45.2) CD4 T cells produced this cytokine compared to the counterparts originating from the transfer (^*^p < 0.05) in *Klrk1*^+/+^ mice (Figure 7C). In contrast, the proportion of endogenous (CD45.2) CD8 T cells producing IFNγ was significantly higher than within transferred CD8 T cells (^**^p < 0.01; Figure 7D). A relatively small but similar proportion of endogenous and transferred CD4 T cells expressed granzyme B (Figure 7C). However, the percentage of CD8 T cells carrying this lytic enzyme was significantly greater in the endogenous cells than in the transferred one (*p* < 0.05, Figure 7D). We observed a significantly greater proportion of transferred (CD45.1) than endogenous (CD45.2) CD4 T cells producing GM-CSF (Figure 7C). Only few or no CD8 T cells produced GM-CSF or IL-17 above background level (data not shown). Notably, elevated proportions of endogenous CD4 and CD8 T cells in the CNS of *Klrk1*^−/−^ recipients produced the immune mediators tested (IFNγ, GM-CSF or granzyme B).

We also measured the expression of NKG2D and could observe that as expected none of the endogenous CD8 T cells carry this receptor in the *Klrk1*^−/−^ recipients. Nevertheless, a significantly higher percentage of endogenous (CD45.2) than transferred (CD45.1) CD8 T cells exhibited NKG2D in wild type recipients. It was not possible to compare transferred and endogenous CD8 T cells in *Klrk1*^−/−^ recipients due to the absence or very low number of CD45.1^+^ CD8 T cells in these mice (Figure 7D). A very low percentage of CD4 T cells expressed NKG2D in our model precluding any comparison. Our results suggest that both endogenous and transferred CD8 T cells could recognize NKG2D ligands in the CNS during passive EAE.

To illustrate the striking differences within CNS-infiltrated T cells, we generated pie charts showing the proportions of cells with an endogenous (CD45.2) or transferred (CD45.1) origin amongst CD4 (Figure 7C) or CD8 (Figure 7D) T lymphocytes. These representations underline that amongst all CNS-infiltrating CD4 T cells those producing IFNγ or GM-CSF originated mainly from the transferred cells (Figure 7C). The second and third column of pies also illustrate the greater proportion of transferred CD4 T cells (CD45.1) producing these cytokines. In contrast, in the CD8 T cell compartment, most cells expressing effector molecules (IFNγ, granzyme B or NKG2D) originated from the endogenous compartment (Figure 7D). In fact, as mentioned above, few transferred CD8 T cells (CD45.1^+^) reached the CNS although similar amount of these cells were co-transferred with CD4 T cells. Nevertheless, most CD45.1^+^CD8 T cells that reached the CNS were able to produce IFNγ and an important proportion expressed NKG2D (Figure 7D second column of pies). Given that a greater proportion of CD8 T cells express NKG2D than CD4 T cells, our results identify CD8 T cells as potential players within the endogenous NKG2D^+^ immune cells contributing to disease severity in the adoptive model of EAE.

## Discussion

In the present study, we establish a novel role for the NKG2D-NKG2DL interaction in the pathobiology of a mouse model of MS. We demonstrate that the activating receptor NKG2D contributes to the pathogenic properties of T lymphocytes that are recruited to the CNS upon peripheral activation of encephalitogenic T lymphocytes. We show that one particular murine NKG2DL, MULT1 is elevated in the target organ and enhances effector functions of activated CD8 T lymphocytes.

Although EAE models do not recapitulate the full complexity of MS pathobiology, they provide relevant *in vivo* models to investigate the contribution of immune factors to different disease stages such as initiation, progression or recovery (31). We investigated the role of NKG2D using these *in vivo* MS models. We induced active EAE in *Klrk1*^−/−^ and *Klrk1*^+/+^ mice from the same breeding colony and noted only a small difference in neurobehavioral scores (Figure 1A). Our results confirm that in the absence of NKG2D, active EAE develops with similar kinetics and amplitude than when this receptor is present. Indeed, Guerra and colleagues observed a slightly reduced EAE disease in *Klrk1*^−/−^ compared to wild type mice only when using a suboptimal dose of MOG_35−55_ (19). It is well established that active EAE is strongly driven by CD4 T lymphocytes. However, NKG2D is only expressed on a small proportion of activated CD4 T lymphocytes (<10% Figure 1C); thus, we can speculate that NKG2D does not alter the encephalitogenic potential of CD4 T lymphocytes in optimal active disease conditions. Ruck and colleagues showed that inhibiting NKG2D after immunization but prior to disease onset reduced disease; however, blocking NKG2D after disease onset had no impact on EAE (17). These authors provided data supporting the idea that blocking NKG2D reduced the migration of T lymphocytes into the CNS. Their results suggest that if NKG2D is blocked while CD4 T lymphocytes that have been activated in response to the immunization are traveling from the periphery to the CNS, it is possible to dampen their capacity to enter this organ. Blocking NKG2D prior to disease onset using soluble NKG2D was more efficient at reducing disease than an anti-NKG2D antibody (17). We can speculate that the soluble NKG2D, in contrast to the anti-NKG2D antibody, could bind to soluble forms of NKG2DL and prevent their effects and accumulation. Overall, these experiments suggest that NKG2D plays a role in CD4 T cell driven active EAE model mainly at disease onset.

To investigate the contribution of NKG2D at the effector phase of EAE and in the absence of adjuvant, we use the passive EAE model. As a greater proportion of activated CD8 T lymphocytes express NKG2D compared to CD4 counterparts (Figure 1D), we optimized our culture conditions to transfer similar proportions of both CD4 and CD8 T cell subsets into naïve recipients. We ruled out any differences in activated wild type lymphocytes between groups and always injected *Klrk1*^−/−^ and *Klrk1*^+/+^ mice in parallel. Disease onset was similar in both groups; however, we reproducibly observed reduced disease severity in *Klrk1*^−/−^ recipients compared to *Klrk1*^+/+^ counterparts for the entire examination period (Figure 6A). Using CD45.1 and CD45.2 detection, we could easily discriminate endogenous leukocytes (CD45.2) from the transferred immune cells (CD45.1) (Figure 7). A greater proportion of CNS-infiltrated T cells originated from the transferred CD4 than CD8 T lymphocytes, regardless of their genotype (Figure 7B). In contrast, endogenous CD8 T lymphocytes were more abundant than the endogenous CD4 T lymphocytes (Figure 7B). This observation is relevant as multiple publications have documented that the number of CD8 T lymphocytes is equal or surpasses the number of CD4 T lymphocytes in MS lesions (32–36). Notably, transferred and endogenous T lymphocytes could produce immune mediators associated with effector functions (IFNγ, GM-CSF, Granzyme B) (Figure 7). Our results support the notion that endogenous immune cells contribute to the disease severity triggered by the transfer of MOG-activated T cells, via a NKG2D-dependent mechanism (Figure 6).

It is well established that the expression of each NKG2DL is regulated at multiple steps in a cell type specific manner (5–7). It is thus necessary to assess the presence of these ligands at the protein level. We established that one NKG2DL in particular, MULT1, was significantly elevated in the CNS at the peak of EAE in both *Klrk1*^−/−^ and *Klrk1*^+/+^ mice while other ligands (Rae-1 and H60) were modestly, or not, altered at the mRNA level (Figure 2A). In line with our results, others also reported elevated mRNA levels of MULT1 in the spinal cord during EAE (30), but they did not assess protein expression. We employed primers detecting all forms of Rae-1 and observed small variations for the expression of this group of NKG2DL (Figure 2). Using specific primers for two Rae-1 family members, Rae1d and Rae1e, Djelloul and colleagues observed elevated mRNA levels in lumbar spinal cord of C57BL/6 mice during EAE (30). Rae-1 was detected at low levels by Western blot in spinal cord of both PLP_139−151_ SJL/J and MOG_35−55_ C57BL/6 EAE mice (37). We detected Rae-1 family members using a pan-antibody by western blot in different CNS areas but did not observe variations between EAE mice and controls (Figures 2B,C). Overall, our data suggest that the protein expression of Rae-1 family members was not significantly modified in the CNS during EAE but we cannot rule out that the expression of specific Rae-1 members was impacted.

In contrast, we established that the protein expression of MULT1 is elevated in the spinal cord at the peak of active EAE disease. Notably, we detected two bands corresponding to the full length (50–55 kDa) and to the cleaved protein (25 kDa) (Figure 3) especially in the spinal cord of EAE mice. The shed form of MULT1 was 4–5 times more abundant than the full length form in spinal cord lysates from EAE animals at the peak of disease (Figure 3). Moreover, we detected an elevated amount of the shed form of MULT1 in the CSF of EAE mice especially at disease peak (Figure 4). We observed similar levels of MULT1 in both *Klrk1*^−/−^ and *Klrk1*^+/+^ groups suggesting that the expression of MULT1 does not depend on the presence of NKG2D on immune cells. The soluble shed form of MULT1 was also detectable in the CSF of passive EAE mice (Figure 6) supporting the notion that neuroinflammatory responses triggered by T cells in the absence of adjuvant also caused MULT1 release in the CSF. Raulet's group detected soluble MULT1 in serum of MULT1^+^ tumor bearing mice and established that the 24 kDa form of MULT1 could be shed via matrix metalloproteinase dependent mechanisms (10). We can speculate that a similar mechanism takes place during EAE as multiple matrix metalloproteinases are elevated in the CNS of EAE mice as well as in the brain tissue, serum and CSF of MS patients (38, 39). Overall, our results show that protein levels of MULT1 are enhanced in the CNS during EAE and that this ligand is present in a shed form detectable in tissue and CSF.

Multiple lines of evidence have established that amongst all NKG2DL, MULT1 exhibits unique properties. MULT1 displays the highest affinity for NKG2D (9). Multiple groups have documented that in mice and humans, shed NKG2DL can act as decoy ligands by preventing NKG2D-expressing immune effector cells from recognizing NKG2DL-bearing target cells, especially in the context of tumor evasion (40, 41). In contrast, the Raulet's group has elegantly shown that shed MULT1 enhances NK cell functions and consequently augments tumor rejection in mice (10). Therefore, we investigated whether soluble MULT1 could similarly enhance T cell functions. In contrast to NK cells, murine T cells do not express NKG2D unless they are activated. To mimic the *in vivo* EAE situation, we activated *in vitro* splenocytes from naïve *Klrk1*^+/+^ and *Klrk1*^−/−^ mice using conditions leading to similar proportion (28–35%) of CD8 T lymphocytes expressing NKG2D in wild type cells (Figure 5A). We added soluble recombinant MULT1, obtained from the same commercial source used by Raulet's group, to the activated splenocytes. We established that the addition of soluble MULT1 upregulated the proportion of CD8 T lymphocytes expressing granzyme B and the cellular expression levels of IFNγ (Figure 5). These boosting effects of soluble MULT1 were not observed in splenocytes from *Klrk1*^−/−^ mice confirming that these effects required NKG2D expression. Our results establish that soluble MULT1 enhances CD8 T cell effector functions via NKG2D. Overall, our *in vivo* and *in vitro* data support the notion that endogenous CD8 T cells contribute to passive EAE pathobiology in a NKG2D-dependent manner and these cells increase their effector functions in response to elevated levels of MULT1. Collectively, our results point to the deleterious role of NKG2D and its ligand MULT1 in the pathobiology of a MS mouse model.

The potential contribution of the NKG2D-NKG2DL interaction to the pathobiology of MS in patients is supported by several publications. Wiendl's group observed that CD4 T lymphocytes carrying NKG2D are enriched in the blood, CSF and post-mortem brain lesions of MS patients compared to control donors especially during relapses (17). Elevated levels of soluble MICB but not MICA have been reported in serum of MS patients compared to healthy donors and even more during relapse compared to remitting phase (15). We have previously shown that human oligodendrocytes both in primary cultures and in post-mortem MS brain tissues express at least one of the NKG2DL (MICA/B) (16). To develop an NKG2DL specific therapy, it will be relevant to investigate the presence of all NKG2DL in MS lesions but also their soluble forms in biological fluids such as CSF and serum. A clinical trial using an anti-NKG2D antagonist antibody in inflammatory bowel diseases has provided the proof of concept that the NKG2D-NKG2DL interaction is a valid therapeutic target in human inflammatory diseases (42). Based on our results, we suggest that directing therapies at one specific NKG2DL instead of the receptor could potentially provide a more specific approach.

## Author Contributions

LL, AM, CL, GD, and SV: conducted experimental work; NA: designed the study; LL, AM, and NA: analyzed and interpreted the data; NA wrote the manuscript and secured funding.

### Conflict of Interest Statement

The authors declare that the research was conducted in the absence of any commercial or financial relationships that could be construed as a potential conflict of interest.
